# Immune resistance and tolerance strategies in malaria vector and non-vector mosquitoes

**DOI:** 10.1186/s13071-017-2109-5

**Published:** 2017-04-18

**Authors:** Tibebu Habtewold, Zoe Groom, George K. Christophides

**Affiliations:** 1 Department of Life Sciences, Imperial College London, London, UK; 2 Department of Comparative Physiology and Biometrics, University of Ghent, Ghent, Belgium; 3 Costello Medical Consulting, Cambridge, UK

**Keywords:** Mosquito immunity, Pathogen tolerance/resistance, *Plasmodium*, Microbiota, Haemolymph antimicrobial activity

## Abstract

**Background:**

The *Anopheles gambiae* complex consists of species that vary greatly in their capacity to transmit malaria. The mosquito immune system has been identified as a key factor that can influence whether *Plasmodium* infection establishes within the mosquito vector. This study was designed to investigate the immune responses of *An. coluzzii*, *An. arabiensis* and *An. quadriannulatus* mosquitoes. The first two mosquito species are major vectors of malaria in sub-Saharan Africa, while the third is thought to be a non-vector.

**Methods:**

All three mosquito species were reared in mixed cultures. Their capacity to eliminate *P. berghei* and regulate midgut bacteria was examined.

**Results:**

Our results revealed large differences in mosquito resistance to *P. berghei*. In all three mosquito species, immune reactions involving the complement system were triggered when the number of parasites that mosquitoes were challenged with exceeded a certain level, i.e. immune tolerance threshold. This threshold was markedly lower in *An. quadriannulatus* compared to *An. coluzzii* and *An. arabiensis*. We also demonstrated that the level of immune tolerance to *P. berghei* infection in the haemolymph is inversely correlated with the level of immune tolerance to microbiota observed in the midgut lumen after a blood meal. The malaria non-vector mosquito species, *An. quadriannulatus* was shown to have a much higher level of tolerance to microbiota in the midgut than *An. coluzzii*.

**Conclusions:**

We propose a model whereby an increased tolerance to microbiota in the mosquito midgut results in lower tolerance to *Plasmodium* infection. In this model, malaria non-vector mosquito species are expected to have increased immune resistance in the haemocoel, possibly due to complement priming by microbiota elicitors. We propose that this strategy is employed by the malaria non-vector mosquito, *An. quadriannulatus*, while *An. coluzzii* has reduced tolerance to bacterial infection in the midgut and consequently reduced immune resistance to *Plasmodium* infection at the haemocoel level. An in-depth understanding of the molecular mechanisms regulating immune tolerance versus resistance in different mosquito vectors of malaria could guide the design of new vector and disease control strategies.

**Electronic supplementary material:**

The online version of this article (doi:10.1186/s13071-017-2109-5) contains supplementary material, which is available to authorized users.

## Background

The mosquito immune system has been identified as a key factor that can influence whether *Plasmodium* infection establishes within the mosquito vector and is the parasite is therefore transmitted to another host. Parasites encounter robust immune responses while invading and developing in the mosquito midgut, which often leads to their elimination. Parasite killing primarily takes place in two major mosquito compartments: the midgut lumen and the haemocoel. Immune responses in the mosquito midgut mainly involve the immune deficiency (*Imd*) pathway and the *NF-κB* transcription factor; *REL2* [1]. The Imd pathway is responsible for maintaining a basal level of immune activity, and its activation relies mostly on recognition of peptidoglycan, a cell wall component found in almost all bacteria. In mosquitoes, and also fruit flies, peptidoglycan shed by the gut microbiota is recognised by peptidoglycan recognition protein LC (*PGRPLC*). This leads to nuclear translocation of *REL2* and production of immune effector molecules [2]. Following a blood meal, bacterial levels increase with a consequent increase of peptidoglycan that enhances the responses of the Imd pathway [3]. Immune effectors of the Imd pathway, synthesised in response to gut bacteria, increase and kill *Plasmodium* parasites that may be present following the ingestion of a blood meal [2]. Additional receptors involved in immune responses to midgut enterobacteria have been revealed, but the signalling pathways through which they function remain unknown [4]. The receptors include three fibronectin type III domain proteins, two of which contain immunoglobulin domains, and a gustatory receptor. The gustatory receptor has been shown to control satiation to sugar meals, providing a link between immune and behavioural responses.

In the haemocoel, immune responses involve humoral reactions of the haemolymph, primarily the complement-like pathway. The *Anopheles* thioester-containing protein 1 (*TEP1*), together with two leucine-rich repeat proteins; *LRIM1* and *APL1*, and several *CLIP*-domain serine protease homologs, play a principal role in the complement-like pathway [5]. *TEP1* and *CLIPs* bind to the surface of *Plasmodium* ookinetes as soon as they appear on the basal sub-epithelial space of the midgut, marking them for lysis and, in some mosquito strains, melanisation [6, 7]. It has been shown that the *TEP1* gene is highly polymorphic in wild *An. gambiae* and *An. coluzzii* populations [8] and that there is an association between certain *TEP1* alleles and resistance to *Plasmodium* [9, 10]. Complement-like pathway responses can also be triggered by bacteria that may be introduced into the hemolymph either artificially [5] or through breaches of the gut epithelium such as those caused by the invasion of malaria parasites [11]. Basal levels of *TEP1*, and presumably other components of this pathway, are also controlled by a signalling pathway orthologous to *Drosophila Toll* that involves the *NF*-*κB* transcription factor, *REL1* [12]. Increased pre-infection levels of *TEP1* have been observed following knockdown of the inhibitor of *REL1* nuclear translocation; *Cactus* [13]. For instance, activation of the *Toll* pathway in *An. gambiae* mosquitoes through the silencing of *Cactus* have been shown to result in increased *P. berghie* killing by *TEP1* [13]. Previous publications have shown that *Cactus* is a negative regulator of *Toll* (*REL1*), which in turn is the upstream regulator of the complement-like pathway [14].

Mosquito hosts respond to pathogen challenges through a combination of immune resistance and tolerance mechanisms. A balance between these two types of response ensures protection from pathogens and, at the same time, limits the collateral damage that may arise as a result of excessive immune effector production. Immune resistance comprises of multiple mechanisms that either prevent pathogens from invading or eliminate pathogens after the invasion has taken place. Immune tolerance mechanisms primarily involve lowered responsiveness to particular immune stimuli and the initiation of processes that protect host tissues from damage caused by the pathogen. This often depends upon the intensity of the challenge by the pathogen. Examples include up-regulation of wound healing and down-regulation of immunopathology, which is often associated with collateral tissue damage, or mitigation of pathogen virulence, through the up-regulation of proteins that can protect host tissues from endotoxins [15–17]. Some molecules have been identified in *Drosophila*, that have been shown to promote tolerance to gut bacteria through the inhibition of the *Imd* pathway [18–20]. In mosquito, for instance, the *Duox-IMPer* (Dual oxidase - Immunomodulatory peroxidase) module is induced after blood-feeding. This facilitates the formation of a dityrosine-linked mucus layer that plugs the ectoperitrophic space (space between the peritrophic membrane and the midgut epithelium) leading to reduced permeability to microbial antigens [21]. As with resistance, mosquito tolerance is believed to be partly regulated by gut microbiota through specialised immune modules, for example, extracellular polysaccharides (EPS). Such molecules block host immune signalling [22–26]. The effect of this blockade varies in magnitude between resistant and susceptible hosts.

Malaria parasites are believed to exploit the interplay between immune tolerance and resistance to establish an infection. Both processes require allocation of resources at the expense of other physiological functions such as survival and reproduction. Mosquito survival and longevity is a key contributing factor to the vectorial capacity of *Anopheles*. This is because only mosquitoes in which the parasite has developed into the sporozoite stage can transmit malaria [27].

We investigate how the balance between immune tolerance and resistance in malaria vector and non-vector species of the *An. gambiae* complex controls *Plasmodium* infection. We reveal that the immune responses in the two main mosquito immune compartments, the midgut and the haemocoel, are inversely related. For example, the level of immune response (tolerance/resistance) in the midgut, often against microbiota, appears to regulate the level of immune response in the haemolymph, possibly due to priming of complement reactions. Malaria non-vector mosquito species show a higher tolerance to gut microbiota, resulting in a greater refractoriness to *Plasmodium* infection, while the opposite is true for malaria vector mosquitoes. The molecular mechanisms regulating immune tolerance versus resistance in different mosquito vectors of malaria could guide the design of new vector and disease control strategies.

## Methods

### Mosquito strain

*Anopheles arabiensis* DONGOLA strain (Sudan, provided by MR4), *An. coluzzii* N’gousso strain (originally *An. gambiae* M form, colonised from field mosquitoes in Yaoundé, Cameroon) and *An. quadriannulatus* SANGQUA strain (Zimbabwe, provided by MR4) were used throughout the experiments.

### Mosquito rearing and maintenance

Mosquitoes were reared and maintained as previously described [28], separately or in mixed cultures. In mixed cultures, equal numbers of L1 stage larvae from each species were pooled together. The individual mosquito species were identified during, or at the end of experiments, using PCR-based methods as previously described [29].

### Infection

A GFP-expressing *P. berghei* ANKA 2.34 strain [28] was used to infect mosquitoes by direct feeding on gametocytemic mice (at 7–8% parasitaemia) as previously described [28] or through the use of membrane feeders using an overnight ookinete culture. The ookinete cultures were first pelleted, and the pellet was then suspended in human blood serum at concentrations of 10^4^, 10^5^, 10^6^, and 10^7^ ookinetes/ml. The average blood meal volume of mosquitoes within the *An. gambiae* complex is about 2 μl (unpublished data); hence each mosquito was infected with approximately 20, 200, 2,000 and 20,000 ookinetes, for the respective dilution. This dose range covers optimum ookinetes to oocyst conversion dose previously reported by [30], i.e. 500 to 3000 ookinete/mosquito.

The parasite infection intensity was determined through direct examination of fluorescent oocysts in the midguts [28] or by quantitative real-time PCR (qPCR). The abundance of the CSP gene fragment ﻿was﻿ measured five days post infection﻿, hence served as a marker for viable oocyst development in the gut.

A modified protocol from [31] was used for *Staphylococcus aureus* culturing and administration for mosquito infection. Briefly, the bacteria culture was allowed to grow to OD_600_ = 0.7 in LB liquid media and then precipitated and washed before suspending in PBS to a final bacterial suspension of OD_600_ = 0.4. Bacteria were injected into the mosquito thorax.

### Midgut dissection

Three types of midgut dissection were carried out: (i) Midguts were removed from female mosquitoes that were infected with *P. berghei* 7 days earlier, and the parasite infection intensity in the gut was quantified by direct examination for fluorescent oocysts as previously described [28]. (ii) Midguts infected with ookinetes using membrane feeder were dissected on day five after infection with *P. berghei*. The infection intensity was determined by measuring the abundance of *P. bergh*ei *CSP* using qPCR on DNA extracted from the midguts. The midgut dissection was carried out on ice cold PBS, and ten midguts were pooled and then transferred into 1.5 ml Eppendorf tubes with tissue lysis buffer and kept on the ice until DNA processing. DNA processing was carried out within two hours of dissection. (iii) For molecular quantification of midgut microbiota, midguts were removed 24 h post-blood meal. Before dissection, the mosquitoes were dipped in sterile water. Forceps used for dissection were sterilised in a flame between each dissection. The dissection was carried out using cold PBS on ice and midguts were then immediately transferred to Eppendorf tubes with tissue lysis buffer.

In each of the above experiments, mosquito carcases were used for species ID.

### dsRNA synthesis and mosquito injection

dsRNA production was performed [28] using gene specific oligonucleotide primers flanked by the short T7 promoter sequence TAA TAC GAC TCA CTA TAG GG. Oligonucleotide primer sequences are shown in Additional file 1: Table S1. For each of the genes, the target region was cloned from a cDNA library, and the dsRNA was synthesised using the MEGAscript T7 Kit (Ambion, Huntington, UK) treated with DNAse I and cleaned using the RNeasy kit (Qiagen, Hilden, Germany). dsRNA concentrations were adjusted to 3 μg/μl. For each dsRNA treatment, 60 female mosquitoes, of two to 3 days old, were placed in a paper cup and injection was performed as previously described [6]. Each mosquito received a volume of 69 ml dsRNA solution injected into the lateral side of the thorax. After 3 days, the mosquitoes were challenged with *Plasmodium* parasite.

Gene silencing experiments using dsRNA were replicated two to three times at an interval of one week or more.

### RT-qPCR analysis

For assessing knockdown efficiency, we measured the level of expression of target genes as described previously [28]. Total RNA was extracted from approximately ten adult mosquitoes using TRIzol reagent (Invitrogen, CA, USA) as described by the protocol. About 1 μg of total RNA (the extraction product) was used for reverse transcription using Superscript II (Invitrogen), which was used in the PCR reaction. For measuring the load of *P. berghei* or microbiota, genomic DNA (gDNA) was extracted from ten pooled midguts using the DNeasy Blood & Tissue kit (Qiagen, Hilden, Germany), and the extraction product was used as a PCR template. The abundance of *P. berghei CSP* and the bacterial 16S rRNA genes were used to detect parasite infection intensity and midgut bacterial load, respectively.

The total volume of reagents was 20 μl containing; 2 μl gDNA or cDNA, 10 μl of 2× SYBR®premix Ex Taq (Takara, Maountain view, USA), 0.2 μM of each primer and 0.4 μl Rox reference dye (50×). Amplification and detection of fluorescence signals were carried out using an Applied Biosystems 7500 Fast Real-Time PCR system. The PCR cycling program consisted of an initial denaturation stage at 95 °C for 20 s, followed by 40 cycles at 95 °C for 3 s and 60 °C for 30 s. Each gene was quantified in duplicate, and the threshold crossing values (C_T_ -values) were standardised (using a standard curve) and normalised to the geometric mean of the S7 rRNA gene that served as an internal control [28].

### Detecting microbiota family

Midgut microbiota families were identified 24 h post-blood meal in each of the three aforementioned mosquito species that were reared in a mixed culture. After dissection, midguts were transferred into separately labelled tubes and kept on ice until the species of mosquito was identified using the PCR method described above. Ten midguts of the same species were pooled, and genomic DNA was extracted using the Wizard® Genomic DNA Purification Kit (Promega, Madison, USA), according to manufacturer’s instructions. Purified gDNA was resuspended in 50 μl of sterile water before PCR amplification. 16S rRNA gene specific primers, 1492r and 27f as described in [32] were used to amplify partial sequences (>750 bp) of a 16S ribosomal gene from bacterial isolates. Products were cloned into the PCR-Blunt Vector and used to transform DH5-alpha cells (Invitrogen). For each mosquito species, 100 white colonies were selected randomly, and the 16S PCR product was sequenced. ClustalW and NCBI blast software was used to identify the bacterial family from the 16S rRNA sequences.

### Haemolymph collection and antimicrobial activity assays

For this experiment, each mosquito species was reared separately as larvae, and the resulting adults were maintained in separate cages. Antimicrobial activity was assessed using a turbidimetry assay in which lytic activity in the haemolymph is determined by the total change in optical density of the aqueous suspension of lyophilized cell wall from *Micrococcus luteus* (*Micrococcus ysodeikticus ATCC* No. 4698, Sigma-Aldrich, Saint Louis, USA). The test was performed as described before [33] with some modifications. First, female mosquitoes were starved for 3 h and were then offered 0.2% phenylthiourea (PTU) in sugar solution. The mosquitoes were then decapitated and immediately transferred to ice cold Millipore Ultrafree®-MC Centrifugal Filter Units of 0.22 μm, containing 60 μl PBS and 0.2% PTU, pH 6.4. The tubes were then spun at 13,000× *rpm* for 30 s, and 20 μl of the hemolymph collection was pipetted into 96-well plates and kept on ice. Lysozyme solution (1 μg/ml) was included as a control and samples were run in triplicate. A 180 μl suspension of *M. luteus* in PBS (0.5 mg/ml) was added to each well. 450 nm absorbance of the substrate-hemolymph mixture was monitored every minute for 60 min using an FLUOstar Omega plate reader. Wells with PBS only were included to control for the decline in turbidity due to settling. The mean change in absorbance of blank samples was subtracted from each experimental reading and the mean change in the Δ*A*_450_ absorbance/minute between mosquito species was compared using an analysis of covariance (ANCOVA).

### Mosquito survival assays

Mosquito daily survival was monitored for 14 days. Survival curves were compared using Log-rank and Gehan-Breslow-Wilcoxon tests. An antibiotic cocktail was used in these assays and included 2 mg/ml Gentamycin (Sigma-Aldrich) and 2 mg/ml Ciprofloxacin (Fluka, Sigma-Aldrich).

### Statistical analysis

Oocyst count data were positively skewed. Thus the Mann-Whitney test was used to compare infection intensity distribution between paired species or gene silenced mosquitoes. Descriptive statistics were calculated for individual replicates. Median and interquartile ranges were calculated and showed a consistent pattern of oocyst distribution between replicates for all three mosquito species. This allowed data to be pooled for statistical analysis.

## Results

### Susceptibility to *Plasmodium*

**Fig. 1:**
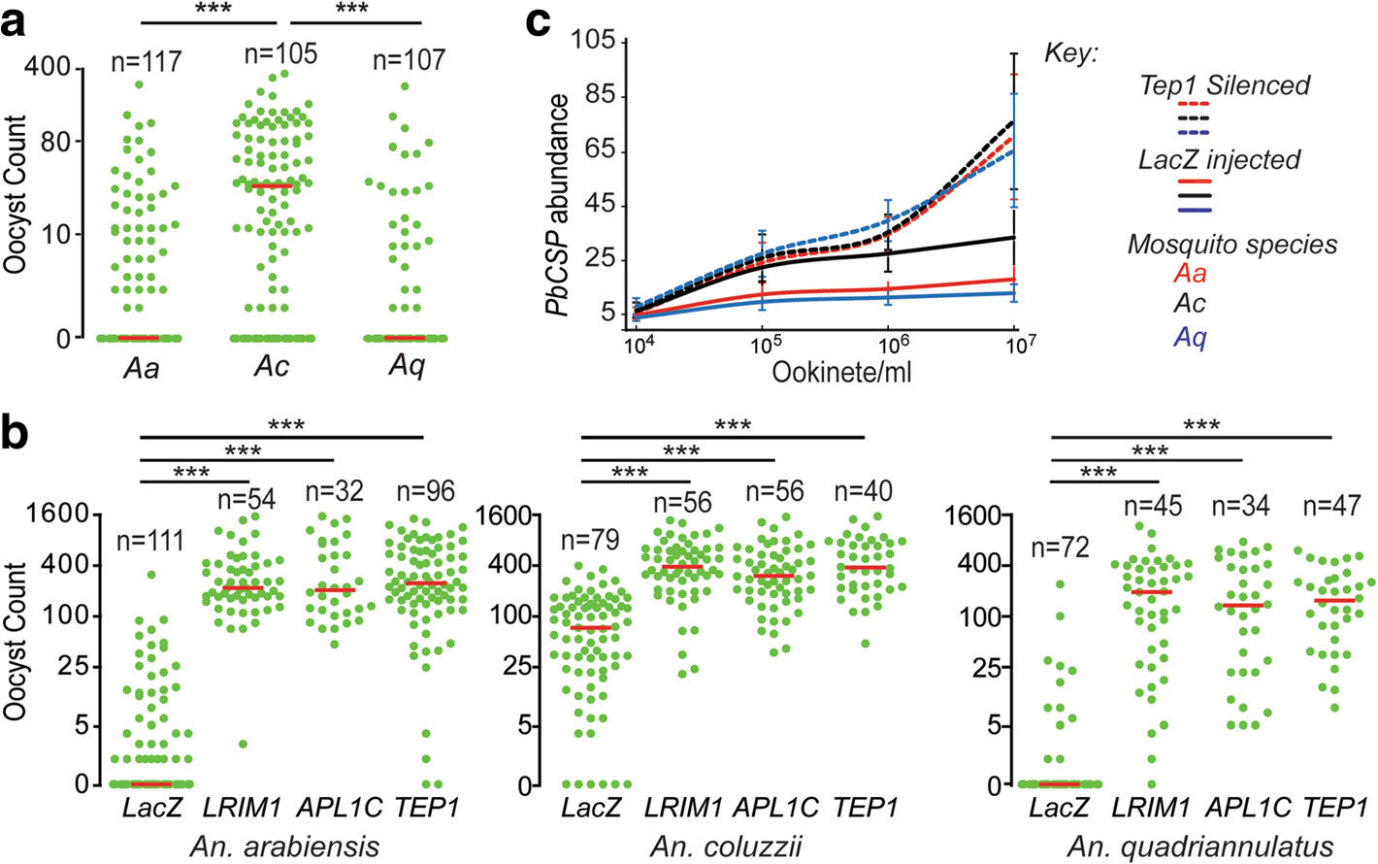
Mosquito infections with *P. berghei* and compliment responses. **a** *P. berghei* oocyst loads in the midguts of *An. arabiensis* (*Aa*), *An. coluzzii* (*Ac*) and *An. quadriannulatus* (*Aq*) pooled from three biological replicates. *Horizontal red lines* show the median oocyst load. Data were analysed by the Mann-Whitney test, and *** corresponds to *P* < 0.001. **b** *P. berghei* oocyst loads following silencing of *TEP1, LRIM1* and *APL1C* or injection with *LacZ* dsRNA (control) in *An. arabiensis*, *An. coluzzii* and *An. quadriannulatus* pooled from two biological replicates. Horizontal red lines show the median oocyst load. Data were analysed by the Mann-Whitney test, and *** corresponds to *P* < 0.001. **c** qPCR analysis of *P. berghei CSP* gene abundance relative to the mosquito S7 gene IN *An. arabiensis* (*red line*), *An. gambiae* (*black line*) and *An. quadriannulatus* (*blue line*) injected with *LacZ* (control) or *TEP1* dsRNA and infected with 10-fold serial dilutions of *P. berghei* ookinetes. The data show a combination of three independent biological replicates

*Plasmodium berghei* infection intensity and prevalence were comparatively investigated in laboratory populations of *An. arabiensis*, *An. coluzzii* and *An. quadriannulatus* mosquitoes that were cultured together from larval stages and genotyped after dissection. A larval co-culturing protocol was used to minimise potential bias arising from variations in mosquito rearing and microbial diversity. The results showed significantly higher oocyst numbers in *An. coluzzii* compared to *An. arabiensis* (U_(18)_ = 2935; *Z* = 50.1, *P* < 0.001) or *An. quadriannulatus* (U_(18)_ = 2170; *Z* = 38.2, *P* < 0.001) (Fig. 1a). In addition, over half of *An. arabiensis* and *An. quadriannulatus* midguts had no detectable oocysts whereas oocyst prevalence was close to 85% in *An. coluzzii*.

*LRIM1*, *APL1C* and *TEP1* were silenced in co-cultured mosquitoes through injection of dsRNA designed against sequences of *An. coluzzii* genes. The infection intensity of *P. berghei* increased drastically in all three mosquito species, and for all three gene knockdowns, compared to *LacZ* dsRNA injected controls (Fig. 1b). No significant difference was observed in the infection intensities (Mann-Whitney test) or prevalence (Chi-square test) between the three species following *LRIM1*, *APL1C* or *TEP1* silencing. In our previous publication, we attained a high and comparable level of gene silencing efficiency for *LRIM1*, *APLC1* and *TEP1 An. coluzzii* and *An. quadiannulatus*, i.e. > 98% reduction of the transcripts of the genes [28]. In that publication, we also reported a high degree of sequence similarity between these species for the exonic region of *LRIM1*, *APLC1* and *TEP1* between the mosquitoes. Comparison of sequences *LRIM1*, *APLC1* and *TEP1* obtained from VectorBase also supports extremely high sequence similarity between these mosquitoes.

We tested the hypothesis that anti-parasitic immune responses in the three mosquito species are differentially triggered when levels of infection exceed a certain threshold. Mosquito species were infected with 10-fold serial dilutions of overnight *P. berghei* ookinete cultures. The abundance of *Pb*CSP transcripts in mosquito midguts was determined after 5 days. *CSP* abundance was similar between the three mosquito species at the starting ookinete dose (10^4^ ookinetes/ml). At this dose, a mosquito receives approximately 20 ookinetes (assuming that the average normal bloodmeal volume of a mosquito within the *An. gambiae* complex is about 2 μl). At the next ookinete dose (10^5^ ookinetes/ml), *CSP* abundance increased unevenly; *An. coluzzii* had higher *CSP* abundance than *An. arabiensis* and *An. quadriannulatus* (Fig. 1c). No significant differences were detected between *An. arabiensis* and *An. quadriannulatus*. When fitted to a logistic distribution model, in all three mosquito populations, the *CSP* transcript levels increased initially but showed an apparent tendency to level off or reach a plateau, at higher concentrations (10^6^ and 10^7^ ookinetes/ml). *CSP* transcript levels were raised by 5, 0.2 and 0.5 fold in *An. arabiensis*, 11.7, 0.4, and 0.4 fold in *An. coluzzii* and 4.5, 0.2 and 0.1 fold following the increase in ookinete dose. Importantly, following *TEP1* silencing, *CSP* transcript levels in all three mosquito species did not increase compared to their respective control mosquitoes at the starting ookinete dose. As the ookinete dose increased, *CSP* transcript levels also increased equally in all three species. These data indicate that, at low levels of infection, all three mosquito species are equally permissive to parasite infection. We, therefore, hypothesise that no/a weak immune response is directed against parasites at this infection level. Conversely, at higher infection levels, mosquitoes mount strong immune responses against parasites, which are more effective in *An. arabiensis* and *An. quadriannulatus,* than in *An. coluzzii* mosquitoes.

### Haemolymph antimicrobial activity

**Fig. 2:**
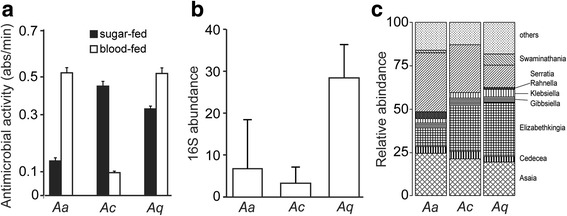
Hemolymph antimicrobial activity and midgut microbiota load. **a** Average antimicrobial activity expressed as change in the absorbance of total body fluids of sugar-fed (*black bar*) and 24-hour blood-fed (*white bar*) *An. arabiensis* (*Aa*), *An. coluzzii* (*Ac*) and *An. quadriannulatus* (*Aq*) female mosquitoes. Analysis of covariance was used to compare regression lines, *P*-values are shown in the text. Error bars show standard error of the mean for data pooled from 10 midguts in two independent biological replicates. **b** Median relative abundance of bacterial 16S rRNA gene measured by qPCR. Error bars represent inter-quartile ranges for data from three independent biological experiments, each consisting ten pooled midguts. **c** Relative abundance of microbiota from the mosquito guts of co-cultured mosquitoes 24 h after blood-feeding

Previous studies have indicated that haemolymph antimicrobial factors, including antimicrobial peptides and complement factors, are produced in response to immune challenge and that these factors also exhibit anti-*Plasmodium* activity [34]. We investigated whether heterogeneity in *Plasmodium* susceptibility between *An. arabiensis, An. coluzzii* and *An. quadriannulatus* is due to inherent differences in the robustness of haemolymph antimicrobial activity. Antimicrobial activity of mosquito haemolymph extracts against the gram-positive bacterium *Micrococcus luteus* was determined using a standard turbidimetric assay. The decline in absorbance was regressed against time and fitted to a model (Fig. 2a). Analysis of covariance (ANCOVA) showed that antimicrobial activity significantly varied among the three mosquito species. When mosquitoes were fed only sugar, significantly higher levels of antimicrobial activity were detected in *An. coluzzii* than in *An. arabiensis* (*r* = 0.98, *P* < 0.001) and *An. quadriannulatus* (*r* = 0.99, *P* < 0.001), while *An. arabiensis* had lower levels of antimicrobial activity compared to *An. quadriannulatus* (*r* = 0.98, *P* < 0.001). The increased antimicrobial activity in the hemolymph of *An. coluzzii* may provide a better immune response to pathogens ingested during sugar feeding.

Antimicrobial activity differed substantially between mosquitoes 24-hours post-blood meal compared to the antimicrobial activity observed in sugar-fed mosquitoes. The antimicrobial activity of hemlymph in blood-fed *An. coluzzii* was significantly reduced compared to sugar-fed *An. coluzzii* (*r* = 0.96, *P* < 0.001), blood-fed *An. arabiensis* (*r* = 0.99, *P* < 0.001) and blood-fed *An. quadriannulatus* (*r* = 0.98, *P* < 0.001). This was in contrast to the antimicrobial activity observed in *An. arabiensis* (*r* = 0.96, *P* < 0.001) and *An. quadriannulatus* (*r* = 0.99, *P* < 0.001), both of which increased significantly in blood-fed mosquitoes compared to sugar-fed controls.

To further investigate differences in antimicrobial activity following a bloodmeal, we monitored the abundance of gut microbiota using qPCR. The bacterial 16S rRNA gene was used as a quantitative marker of bacterial abundance in co-cultured *An. arabiensis*, *An. coluzzii* and *An. quadriannulatus*. Analysis of qPCR results showed that *An. quadriannulatus* had a 4-fold higher microbial load than *An. arabiensis* and a 9-fold higher load than *An. coluzzii* (Fig. 2b). Microbial numbers were 2-fold higher in *An. arabiensis* than in *An. coluzzii* and this difference were not significant.

Sequencing of 100 16S rRNA PCR products from pools of ten mosquitoes for each of the co-cultured species identified similar microbiota families to those previously reported in the *An. coluzzii* laboratory colony [35], including *Asaia* sp*.*, *Cedecea* sp*.*, *Elizabethkingia* sp*.*, *Gibbsiella* sp*.*, *Klebsiella* sp*.*, *Rahnella* sp*.*, and *Serratia* sp*.* There were no major differences between the three mosquito species for the major microbiota families (Fig. 2c).

### Mosquito longevity

**Fig. 3:**
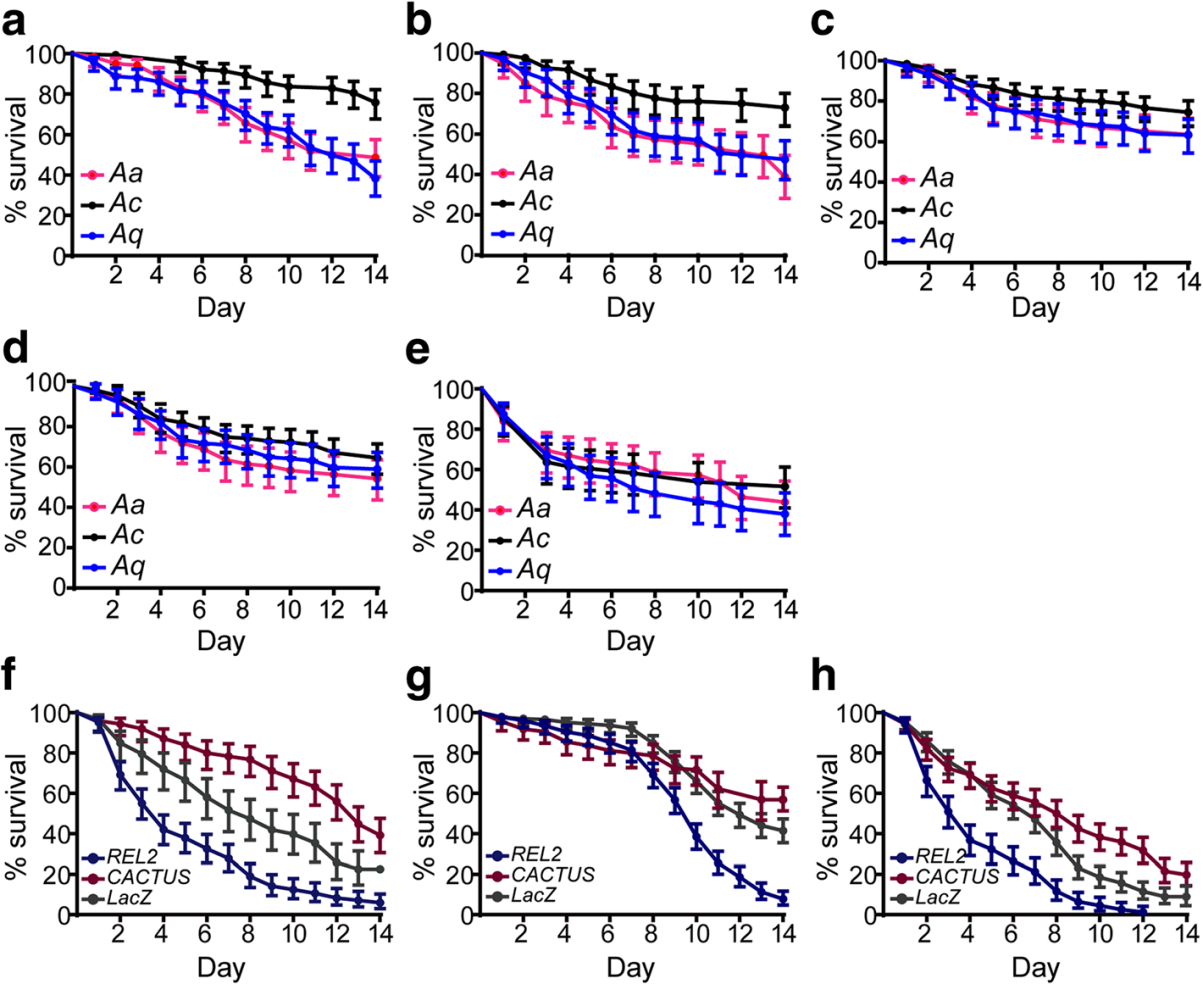
Daily survival of adult mosquitoes*.* **a**-**c** Survival curves of adult *An. arabiensis* (*Aa*), *An. coluzzii* (*Ac*) and *An. quadriannulatus* (*Aq*) female mosquitoes maintained on sugar solution (**a**), obtained a single bloodmeal from non-infectious mice on day 3 post-emergence (**b**) or maintained on sugar solution supplemented with antibiotics for the first 3 days post-emergence (**c**). Survival curves of adult female mosquitoes infected with *P. berghei* parasite (**d**) by direct feeding on gametocytemic mice (at 7–8% parasitaemia) or injected with *S. aureus* (**e**). **f**-**h** Survival curves of adult female *An. arabiensis* (**f**), *An. coluzzii* (**g**) and *An. quadriannulatus* (**h**) injected with *LacZ* (control), *REL2* and *Cactus* dsRNA. Error bars represent 95% CI for three independent biological replicates

Host immune competence and survival are interrelated and are both important components of host fitness. We monitored the survival rates of mosquito populations to examine the impact of immune responses on fitness. Survival assays were carried out on adult females reared together since initial larval stages using the co-culturing protocol (Fig. 3). Mosquitoes were subject to the following treatments: (a) control, maintained on sugar solution throughout the experiment, (b) blood-fed on naïve mice 3 days post-emergence, and (c) maintained throughout on a sugar solution containing an antibiotic cocktail. A significantly lower survival rate was detected in sugar-fed *An. arabiensis* (48.7%, *χ* ^2^ = 23.9, *df*  = 1, *P* < 0.001) and *An. quadriannulatus* (38.3%, *χ* ^2^ = 37.6, *df*  = 1, *P* < 0.001) mosquitoes compared to *An. coluzzii* (75.8%) (Fig. 3a). These differences did not change after mosquitoes received a blood meal, i.e. for *An. coluzzii* (72.9%) *vs* *An. arabiensis* (38.9%, *χ* ^2^ = 21.5, *df*  = 1, *P* < 0.001); *An. coluzzii vs An. quadriannulatus* (47.4%, *χ* ^2^ = 14.5; *df * = 1; *P* < 0.001) (Fig. 3b). However, the survival rates of *An. quadriannulatus* (63.5%) and *An. arabiensis* (65.2%) increased when mosquitoes were treated with antibiotics (Fig. 3c). Survival rates were highest in *An. coluzzii* (74.5%). These data suggest that the high gut microbiota loads in *An. quadriannulatus* compared to *An. coluzzii* may negatively impact on mosquito survival.

We investigated the effect of infection on mosquito longevity by monitoring the survival rates of the three species under the following treatments: (d) blood-feeding on *P. berghei* infected mice 3 days post-emergence, and (e) infection (*via* hemolymph inoculation) with *Staphylococcus aureus* bacteria (unknown lab strain) 3 days post-emergence. The results showed that differences in survival rates between the mosquito species were removed when they were challenged with *P. berghei* or *S. aureus*, respectively (Fig. 3d, e). The survival rates for *P. berghei* challenged *An. coluzzii, An. arabiensis* and *An. quadriannulatus* were 65.4, 57.7 and 59.4%, respectively, and survival rates after *S. aureus* infection were 52.1, 44.0 and 38.7%. This effect is most likely due to the life-shortening effect of pathogens on *An. coluzzii*. Taken together, these data suggest that high levels of hemocoel immunity, influenced by an increased abundance of gut microbiota, which is related to reduced epithelial immune response (increased tolerance to bacteria), can be beneficial to mosquitoes during infection but detrimental in the absence of infection. The converse is true for reduced levels of hemocoel immunity (tolerance levels), which impact mosquitoes positively in the absence of infection but negatively during infection.

A significant increase in the proliferation of bacteria has been observed in *REL2* silenced *An. gambiae* mosquitoes, but the microbial level was less when *REL1* mediated *Cactus* expression was silenced. To test the hypothesis that mosquito immune competence and survival are interrelated, we knocked down in co-cultured mosquito populations the *NF-κB* transcription factor *REL2* and thus silenced the antibacterial *Imd* pathway [36], and the negative regulator of *REL1*, *Cactus* [13]. As a result, we ectopically activated the Toll pathway that shares most of its downstream effectors with those of the *Imd* pathway and is thought to be antifungal (unpublished data). Mosquitoes injected with *dsLacZ* served as controls.

A similar and significant silencing efficiency was achieved in all three mosquito species: 60–64% for *REL2* and 78–82% for *Cactus*. The level of 16S rRNA gene was not measured in *REL2* and *Cactus* silenced mosquitoes, because we expect similar results as the previously published observations by [2] with these genes/pathways on the bacterial in the midguts. Over the monitoring period, silencing *REL2* significantly decreased survival, in *An. arabiensis* (5.9%, *χ* ^2^ = 26.0, *df*  = 1, *P* < 0.001), *An. coluzzii* (7.8%, *χ* ^2^ = 78.9, *df*  = 1, *P* < 0.001) and *An. quadriannulatus* (0%, *χ* ^2^ = 44.2, *df*  = 1, *P* < 0.001) compared to the *LacZ* treatments in all three populations (Fig. 3 f-h). The survival rate, in the respective mosquito species, was 22.4, 41.5 and 8.9% for *LacZ* treatment. Interestingly, the effect of *REL2* silencing on *An. coluzzii* became apparent only late in mosquito life (Fig. 3g). This confirms the functional importance of the *Imd* pathway for mosquito survival. *LacZ* treated group*, Cactus* silencing significantly increased survival of *An. arabiensis* (42.8%) (*χ* ^2^ = 15.3, *df*  = 1, *P* < 0.001) and *An. quadriannulatus* 19.8% (*χ* ^2^ = 13.5, *df* = 1, *P* < 0.001) but not *An. coluzzii* mosquitoes (56.8%). The survival curves for *Cactus* and *REL2* silenced mosquitoes was significantly different for each mosquito species (*χ* ^2^ = 94.1, *df * = 1, *P* < 0.001 for *An. arabiensis*; *χ* ^2^ = 63, *df*  = 1, *P* < 0.001 for *An. coluzzii*; and *χ* ^2^ = 78.2, *df*  = 1, *P* < 0.001 for *An. quadriannulatus*). These data suggest that compromising the mosquito antibacterial immune response has a deleterious effect on survival, even when mosquitoes are not additionally challenged with a pathogen. Boosting basal levels of immunity before infection provides a fitness advantage to *An. arabiensis* and *An. quadriannulatus* but not *An. coluzzii*.

## Discussion

Differences in vectorial capacity between sibling mosquito species of the *An. gambiae* complex provides an excellent framework to investigate factors contributing to phenotypes associated with *Plasmodium* infection and malaria transmission. In this study, experiments were conducted using a mosquito management protocol that minimises possible biases introduced during mosquito rearing, maintenance and handling. *Anopheles arabiensis*, *An. coluzzii* and *An. quadriannulatus* mosquitoes were reared in a mixed culture starting from first-instar larva (L1) and maintained together until completion of the experiments conducted during adulthood.

We confirmed that between the three mosquito species, *An. coluzzii* is the most permissive to infection with *P. berghei*, while *An. quadriannulatus* is the most refractory. This is consistent with findings of previous studies that used both *P. berghei* and *P. falciparum* [28, 37]*.* We have also revealed that *An. arabiensis* is highly refractory to *P. berghei*. This contrasts with the high Victorial Capacity in the *An. arabiensis* that have been reported across sub-Saharan Africa and also previous observations made in field experiments in West Africa indicating that the mosquito species is as susceptible to *P. falciparum* as *An. coluzzii* [38]*.* One of the possible explanations for this discrepancy is that these two sibling African vectors of human malaria have adapted to have increased tolerance to *P. falciparum* as a result of their sympatric co-evolution [39]. In addition, a recent study has revealed that genetic variation within mosquito populations can greatly affect their susceptibility to *Plasmodium* infection [40].

Our data show that resistance to *Plasmodium* infection in *An. arabiensis* and *An. quadriannulatus* is mediated by the mosquito immune system. Both species become highly and equally susceptible to infection when the expression of key molecules of the complement pathway are silenced. These data support previous findings showing that *Plasmodium* invasion of the mosquito midgut is largely limited by reactions of the complement pathway in *An. coluzzii* mosquitoes [6, 28, 41]. Importantly, neither susceptibility nor resistance to parasites was fully penetrant within the three populations. These data, in conjunction with the finding that all three populations demonstrated similar infection phenotypes following complement knockout, suggest the presence of genetic variation associated with the infection phenotype of each population. Indeed, we have been able to genetically select both a highly permissive and a highly refractory *An. arabiensis* line within a limited number of generations (data not shown).

All three mosquito species appear to be equally permissive to *P. berghei* infection at low infection rates*.* We have shown that oocyst loads in the midguts of *An. arabiensis, An. quadriannulatus* and *An. coluzzii* are very similar after challenge with a small number of parasites and are not affected by silencing of the complement pathway. These data suggest that complement reactions are not triggered at low infection rates. Infection intensity dependent responses have been previously reported for *An. coluzzii* infected with natural populations of *P. falciparum* [42]. Furthermore, reduced differences in susceptibility to *P. berghei* among *An. stephensi* mosquitoes of a laboratory colony have been previously reported at low infection intensities [43]. Similar observations have been made for *Onchocerca* roundworm infections of blackflies [44].

Differences in infection intensities between the three mosquito species become significant as the parasite challenge increases. At a dose of approximately 200 ookinetes/mosquito, infection intensities reach a plateau in *An. arabiensis* and *An. quadriannulatus* and further dose increase cause little or no change unless the complement system is inactivated. This level of tolerance is ten times higher in *An. coluzzii* (higher by approximately 2000 ookinetes per mosquito). Previous plant and animal studies have demonstrated the existence of variation in tolerance to pathogenic infections in different breeds, strains or individuals [45, 46]. Together, our data indicate that differences in susceptibility to parasite infection between the three closely related mosquito species are due to different levels of immune tolerance. The immune responses are activated in all three species as soon as the respective levels of immune tolerance are breached when the pathogen challenge passes a respective threshold.

Basal levels of mosquito immune response are thought to be modulated by midgut microbiota that activates immune signalling pathways leading to the production of antimicrobial effectors [31, 34, 47, 48]. An increase in microbial loads, such as that observed after blood-feeding, further increases the levels of antimicrobial effectors. These effectors also have anti-parasitic activities. The haemolymph antimicrobial activities of *An. quadriannulatus* and *An. arabiensis* 24 h after blood-feeding dramatically increases, reaching much higher levels than *An. coluzzii*. At the same time, their gut microbiota loads were higher than those of *An. coluzzii*. These data suggest the presence of 2 distinct immune compartments; the midgut lumen and the haemocoel, each with an opposite level of immune tolerance or resistance to the other. Note worth that the haemolymph antimicrobial activity was measured as the rate of digestion of cell wall from gram-positive bacterium *Micrococcus luteus.*

In this present work, midgut microbiota was not measured in the *REL2* and *Cactus* KD *An. arabiensis* and *An. quadriannulatus* mosquitoes because we assume that there are no major departures from previously published observations in *An. gambiae* [2]. The authors demonstrated that the abundance of microbiota increased significantly in *REL2* KD *An. gambiae* mosquitoes compared to *LacZ* injected mosquitoes, but there was a significant reduction in the microbial number in Cactus KD mosquitoes.

**Fig. 4:**
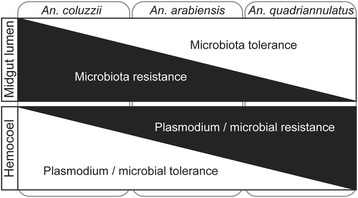
Model of immune tolerance and resistance equilibrium. The model depicts two immune compartments, the midgut lumen and hemocoel, separated by the midgut epithelium. A highly efficient malaria vector, *An. coluzzii* (*Ac*), and non-vector *An. quadriannulatus* (*Aq*) are placed at different areas of the model by the observed interplay between the tolerance and resistance in the two immune compartments

Our data lead us to propose the following model of immune tolerance versus resistance in malaria vector *An. coluzzii* non-vector *An. quadriannulatus* investigated in this study (Fig. 4). The two mosquito species follow distinctly different mechanism of immune response. *An. quadriannulatus* has elevated levels of immune response at the midgut level compared to *An. coluzzii*, which leads to high gut microbial load following blood-feeding. These high microbial loads are associated with elevated levels of bacterial-derived immune elicitors such as peptidoglycan that trigger activation of the Imd and possibly other immune pathways, both locally in the gut and systemically in the haemocoel. Immune pathway activation in the gut aims to control bacterial levels and restore homoeostasis, while systemic immune responses in the haemocoel aim to prevent pathogens from breaching the epithelial barrier and establishing infection. The latter leads to elevated levels of resistance against *Plasmodium* parasites, which approximately 24 h after ingestion, penetrate the midgut epithelium to establish an infection on the basal sub-epithelial space that is an extension of the haemocoel. On the other hand, *An. coluzzii* exhibits a low level of tolerance to gut microbiota, which in turn leads to low haemolymph antimicrobial activity and increased tolerance to *Plasmodium* invasion. Intriguingly, the haemolymph antibacterial activity in *An. coluzzii* reduced drastically following a blood meal. This immune suppression may be part of the energy trade-off between the two immune compartments, whereby increased resistance to bacterial proliferation in the midgut after a blood meal is linked to immune repression and increased tolerance in the haemolymph. Indeed, it has been suggested that susceptibility to *Plasmodium* infection in *An. gambiae* is a result of purposeful immune suppression to prevent over-activation of immune response following ingestion of a blood meal [21, 48]. An example of such a response is the formation of ImPer-DUOX, a protein network that creates a barrier to restrict midgut-derived microbial immune elicitors from reaching immune receptors on epithelial cells. *An. arabiensis* appears to show moderate tolerance and resistance levels in both compartments.

Excessive or uncontrolled immune responses can have an adverse impact on fitness and reproduction. Female mosquitoes require a blood meal to obtain the necessary nutrients and energy for reproduction, but this is associated with increased risk of infection from blood-borne pathogens or their microbiota. The tolerance versus resistance model that we have proposed for the three mosquito species may represent different configurations of a resource management strategy that is in conjunction with accommodation of parasitism with *Plasmodium*. The malaria non-vector, *An. quadriannulatus,* tolerates an increase in bacteria after a blood meal but upregulates immune response in the haemocoel in anticipation of potential pathogen invasion, thus restricting *Plasmodium* infection. Conversely, the malaria vector, *An. coluzzii,* has evolved to resist bacterial over-proliferation in the gut lumen and therefore limits haemolymph responses, perhaps as a result of its co-evolution and co-adaptation with *Plasmodium*. The strategy of *An. coluzzii* appears to be more cost effective in the absence of *Plasmodium* infection, as demonstrated by the fact that this mosquito was shown to live longer. However, in the presence of infection, its survival is compromised, possibly due to gut bacteria that take advantage of epithelial invasion by *Plasmodium* to infect the non-immune haemolymph [49].

Beyond their significance in understanding immune response management and homoeostasis in mosquitoes, our data reveal new opportunities for exploring potential targets for developing novel vector and malaria control tools. Targeting tolerance or resistance to microbiota in the mosquito gut could be exploited towards modifying the mosquito immune response against *Plasmodium* in the haemocoel and inducing premature death due to the collateral damage of internal organs. It is plausible to achieve total *Plasmodium* transmission blockade using transgenic mosquito approaches. Using this approach, the mosquito immune system can be designed to augment parasite killing by targeting mosquito anti-malarial regulators that function upon binding to the parasite surface, for example, *SPCLIP1* that binds to the ookinete and facilitates complement attacks [5]. Alternatively, the premature killing of mosquito vectors, before the parasite completes the gonotrophic cycle, can be achieved through transgenic approaches. This includes the constitutive knockout or overexpression of bacterial receptors that disrupt gut homeostasis and eventually kill the mosquito due to opportunistic infections or altered physiology. Some receptors, with a significant role as regulators of gut antibacterial defences, have been identified. These include *PGRPLC*/A, fibronectin type-III domain proteins (*FN3D1*-3) and a gustatory G-protein coupled receptor (*GPRGR9*), which can be exploited to reduce mosquito longevity.

## Conclusion

The level of immune response (tolerance/resistance) in the midgut, often against microbiota, appears to regulate the level of immune response in the haemolymph, possibly due to priming of complement reactions. Malaria non-vector mosquito species, An. quadriannulatus, show a higher tolerance to gut microbiota, resulting in a greater refractoriness to Plasmodium infection, while the opposite is true for malaria vector mosquitoes. An in-depth understanding of the molecular mechanisms regulating immune tolerance versus resistance in different mosquito vectors of malaria could guide the design of new vector and disease control strategies.

## Additional file

Additional file 1: Table S1. Oligonucleotide primers used in gene abundance measurements and silencing. (DOCX 16 kb)

## Abbreviations

APL1: *Anopheles*-*Plasmodium*-responsive leucine-rich repeat 1
CSP: Circumsporozoite protein
Duox-IMPer: Dual oxidase - immunomodulatory peroxidase
GFP: Green fluorescent protein
Imd: Immune deficiency pathway
NF-κB: Nuclear factor-kappaB
PGRP: Peptidoglycan recognition protein
qPCR: Quantitative real-time polymerase chain reaction
RLIM1: Leucine-rich repeat proteins 1
RT-qPCR: Reverse-transcription qPCR
TEP1: Thioester-containing protein 1
