# Supplementary material for: Immune resistance and tolerance strategies in malaria vector and non-vector mosquitoes
Source: Parasit Vectors. 2017 Apr 18;10:186. doi: 10.1186/s13071-017-2109-5 (PMC5395841; doi:10.1186/s13071-017-2109-5)
Supplement: Additional file 1: Table S1. — Oligonucleotide primers used in gene abundance measurements and silencing. (DOCX 16 kb) [file 13071_2017_2109_MOESM1_ESM.docx]

**Additional file 1: Table S1.** Oligonucleotide primers used in gene abundance measurements and silencing

| Gene name | Gene ID | Forward primer | Reverse Primer |
| --- | --- | --- | --- |
| Gene abundance | | | |
| *S7* | AGAP010592 | GTGCGCGAGTTGGAGAAGA | ATCGGTTTGGGCAGAATGC |
| *16S rRNA* | n/a | CTCCTACGGGAGGCAGCAG | GAATTACCGCGGCKGCTG |
| *PbCSP* | PBANKA_040320 | GAATTCGTTAAACAGATCAGGGATAGTA | TTATACCAGAACCACATGTTACGTTACA |
| Gene silencing | | | |
| *LacZ* | n/a | GAATTAATACGACTCACTATAGGGAGAATCCGACGGGTTGTTACT | GAATTAATACGACTCACTATAGGG CACCACGCTCATCGATAATTT |
| *CACTUS* | AGAP007938 | GAATTAATACGACTCACTATTAGGGAGAGTCCGCTCTACACATCAGCA | GAATTAATACGACTCACTATTAGGGAGACCGTTCGGGTTAATGATGAC |
| *REL2* | AGAP006747 | GAATTAATACGACTCACTATTAGGGAGAAATCCGACGCAAAGATACG | GAATTAATACGACTCACTATTAGGGAGAGACCGCAATGTGAAGGATG |
| *LRIM1* | AGAP006348 | GAATTAATACGACTCACTATAGGGAATATCTATCTCGCGAACAATAA | GAATTAATACGACTCACTATAGGGTGGCACGGTACACTCTTCC |
| *APL1C* | AGAP007033 | GAATTAATACGACTCACTATAGGGGCTTACGCGCACACTATTCA | GAATTAATACGACTCACTATAGGGGCTATTGTGCGATGCGTCTA |
| *TEP1* | AGAP803155 | GAATTAATACGACTCACTATAGGGTTTGTGGGCCTTAAAGCGCTG | GAATTAATACGACTCACTATAGGGACCACGTAACCGCTCGGTAAG |
